# The Asp^298 ^allele of endothelial nitric oxide synthase is a risk factor for myocardial infarction among patients with type 2 diabetes mellitus

**DOI:** 10.1186/1471-2261-8-36

**Published:** 2008-12-10

**Authors:** Jacob Odeberg, Charlotte A Larsson, Lennart Råstam, Ulf Lindblad

**Affiliations:** 1 Department of Medicine, Atherosclerosis Research Unit, Centre for Molecular Medicine, Karolinska Institutet, University Hospital, Stockholm, Sweden; 2 Karolinska Biomics Centre, Karolinska University Hospital, Stockholm, Sweden; 3 Department of Clinical Sciences, Malmö, Community Medicine, Lund University, Malmö University Hospital, Malmo, Sweden; 4 Department of Public Health and Community Medicine/Primary Health Care, The Sahlgrenska Academy at Gothenburg University, Sweden; 5 Skaraborg Institute, Skövde, Sweden

## Abstract

**Background:**

Endothelial dysfunction plays a central role in atherosclerotic progression and cardiovascular complications of type 2 diabetes mellitus (T2DM). Given the role of nitric oxide in the vascular system, we aimed to test hypotheses of synergy between the common endothelial nitric oxide synthase (eNOS) Asp^298 ^allele and T2DM in predisposing to acute myocardial infarction (AMI).

**Methods:**

In a population-based patient survey with 403 persons with T2DM and 799 healthy subjects from the population without diabetes or hypertension, we analysed the relation between T2DM, sex and the eNOS Asp^298 ^allele versus the risk for AMI.

**Results:**

In an overall analysis, T2DM was a significant independent risk factor for AMI. In patients with T2DM, homozygosity for the eNOS Asp^298 ^allele was a significant risk factor (HR 3.12 [1.49–6.56], p = 0.003), but not in subjects without diabetes or hypertension.

Compared to wild-type non-diabetic subjects, all patients with T2DM had a significantly increased risk of AMI regardless of genotype. This risk was however markedly higher in patients with T2DM homozygous for the Asp^298 ^allele (HR 7.20 [3.01–17.20], p < 0.001), independent of sex, BMI, systolic blood pressure, serum triglycerides, HDL -cholesterol, current smoking, and leisure time physical activity. The pattern seemed stronger in women than in men.

**Conclusion:**

We show here a strong independent association between eNOS genotype and AMI in patients with T2DM. This suggests a synergistic effect of the eNOS Asp^298 ^allele and diabetes, and confirms the role of eNOS as an important pathological bottleneck for cardiovascular disease in patients with T2DM.

## Background

Diabetes constitutes one of the major independent cardiovascular risk factors, and patients with this disease suffer from premature cardiovascular morbidity and mortality. Endothelial dysfunction is regarded as an early step in the development of insulin resistance and T2DM as well as important for the atherosclerotic predisposition and cardiovascular complications associated with diabetes [1]. Endothelial Nitric Oxide (NO) is a critical actor in the pathophysiology of the vascular system [1-3] and is involved in preventing the oxidation of lipoproteins, in down-regulating inflammatory mediators, preventing the adhesion of monocytes to the endothelium, controlling the expression of proteins involved in atherogenesis, and in inhibiting the proliferation of vascular smooth muscle cells [1,2]. Furthermore, it has a protective effect in angina and the acute coronary syndrome by promoting vessel vasodilation and by inhibiting platelet adhesion and aggregation [1,2,4]. A reduced bioavailability of NO may result from down-regulated protein expression, depressed activation, or reduced enzymatic function of endothelial nitric oxide synthase (eNOS) or from increased consumption and inactivation of the NO produced [2,5,6]. Elevated glucose, smoking, inflammation and oxidative stress result in the production of reactive oxygen species (ROS) that directly can scavenge NO [2,7]. Several risk factors for coronary artery disesase (CAD) such as smoking, elevated homocysteine, free fatty acids, hypertension, insulin resistance, and diabetes also depress eNOS [2,6,8].

The Glu298Asp polymorphism is the only coding region variant identified in the eNOS gene and has been associated with an increased risk of ischemic heart disease (IHD) [9,10]. Findings suggest that subjects carrying the Asp^298 ^allele generate lower NO *in vivo *and could be more susceptible to endothelial dysfunction [11], however the genotype effect may be restricted to endothelial cells as results indicate that there is no direct effect of the polymorphism on NO production in platelets [12]. Other factors which reduce NO bioavailability could hypothetically act in synergy with the Asp^298 ^allele and precipitate conditions of inadequate NO bioavailability. This could result in endothelial vulnerability and a more prothrombotic state in the case of endothelial damage or plaque rupture. In this study, we aimed to test this hypothesis by focusing on a possible synergistic effect of T2DM and the eNOS Asp^298 ^allele with respect to AMI morbidity.

## Methods

### Subjects

In Skara, a small community in the Skaraborg County in southwestern Sweden, patients with hypertension and/or diabetes have been treated at special outpatient clinics within primary care since the 1970s (The Skaraborg Hypertension Project) [13]. From 1992 to 1993, all 1149 patients with hypertension and/or T2DM who completed an annual check-up at the hypertension and diabetes clinic in Skara were consecutively surveyed for cardiovascular risk factors (The Skaraborg Hypertension and Diabetes Project) [14,15]. From 1993 to 1994, a population survey using the same protocol as the patient survey was conducted with a randomized sample from the population census register, stratified by age and sex [16]. Out of 1400 invited subjects = 40 years of age, 1109 (80%) completed the study visit. Among the patients, 454 subjects were diagnosed with diabetes. After the exclusion of patients with type 1 diabetes (n = 33), missing blood samples (n = 12), and unsuccessful genotyping (n = 6), 403 patients remained for further analyses in the current study. In the population survey of 1109 persons, we identified 824 participating subjects who neither had hypertension nor diabetes [17] that was used as a reference group. Previous cardiovascular event was not an exclusion criteria. Out of these, 799 subjects were successfully genotyped. The characteristics of the study population are shown in table 1.

**Table 1:** Descriptive characteristics of the study population

	**T2DM**				**Controls**			
	**Men**		**Women**		**Men**		**Women**	
	(n = 202)		(n = 201)		(n = 391)		(n = 408)	
Variables (Units)	Mean	(SD)	Mean	(SD)	Mean	(SD)	Mean	(SD)
Age (years)	69.4	(9.3)	70.7	(10.2)	60.8	(13.1)	59.5	(12.4)
Waist hip ratio (WHR) (cm)	0.96	(0.06)	0.88	(0.07)	0.93	(0.08)	0.81	(0.08)
Body Mass Index (BMI) (kg m^-2^)	27.9	(3.7)	28.9	(4.9)	26.0	(3.6)	26.0	(4.7)
Systolic blood pressure (mm Hg)	154	(18.5)	154	(18.6)	133	(18.2)	136	(18.1)
Diastolic blood pressure (mm Hg)	86	(9.7)	83	(10.4)	76	(9.5)	74	(10.1)
Triglycerides (mmol/L)	1.6	(0.9)	1.6	(0.8)	1.2	(0.8)	1.1	(0.8)
Total Cholesterol (mmol/L)	5.7	(1.1)	6.0	(1.2)	5.9	(1.1)	6.1	(1.2)
LDL Cholesterol (mmol/L)	3.9	(1.0)	4.1	(1.1)	4.3	(1.0)	4.3	(1.1)
HDL Cholesterol (mmol/L)	1.0	(0.23)	1.1	(0.25)	1.1	(0.24)	1.2	(0.24)
								
	**n**	**(%)**	**n**	**(%)**	**n**	**(%)**	**n**	**(%)**
Smoking (yes or no)	41	(25.0)	27	(28.2)	83	(22.2)	76	(17.5)
High leisure time physical activity	27	(10.6)	8	(4.9)	122	(32.9)	81	(19.6)
Glu298Glu	112	(55.5)	110	(54.7)	195	(49.9)	212	(52.0)
Glu298Asp	75	(37.1)	78	(38.8)	157	(40.1)	165	(40.4)
Asp298Asp	15	(7.4)	13	(6.5)	39	(10.0)	31	(7.6)

Data are means and (SD), or numbers (n) and (%). Means were adjusted for age difference and proportions were standardised to the population in Skara. Genotype distributions were in HW. HDL, High-Density Lipoproteins;

LDL, Low-Density Lipoproteins; LTPA; leisure time physical activity.

The regional ethical review board at Gothenburg University, Gothenburg, Sweden, approved both surveys. Subjects received both written and oral information and were included after giving consent. The study was carried out in accordance with the principles of the Declaration of Helsinki.

### Methods

Procedures in this project have been published in detail previously [14-18]. Participants were seen in the morning after an overnight fast (10 h) and venous blood samples were drawn. Body weight and height were measured in light clothes and no shoes. Blood pressure was measured in a supine position after 5 minutes rest (arm in heart level and cuff size adjusted for arm circumferences) [14]. Information on medical history and ongoing medication was collected and participants answered a validated questionnaire on smoking habits, alcohol consumption, and physical activity [14]. Current smoking was defined as daily smoking (yes/no). Leisure time physical activity (LTPA) was divided into those who exercised (walking and gardening included) for at least 4 hours per week in their leisure time, and those who did not [17]. BMI was calculated by weight (kg) × length^-2 ^(m). Serum triglycerides and HDL-cholesterol were analyzed using standard commercial kits.

In accordance with national and international guidelines at the time, diagnosis of hypertension was based on three consecutive readings of diastolic blood pressure ≥ 90 mm Hg, or ongoing treatment with blood pressure lowering medications for hypertension [19]. Diagnosis of diabetes was based on two fasting blood glucose values ≥ 6.7 mmol L^1^, a 2 hour blood glucose value of ≥ 11.1 mmol L^-1 ^in an oral glucose tolerance test (OGTT), or a known diagnosis of diabetes [20]. Differentiation between type 1 and T2DM was based on clinical criteria.

The end-point register in the project includes fatal or non-fatal first events of AMI and acute stroke (morbidity), all fatal AMI and acute stroke events (mortality), and all-cause mortality. Information on end-points, from baseline through 2002, was ascertained by record linkage with the Swedish national mortality and in-patient registers. According to our previous evaluation this method is valid [21]. With few exceptions, AMI events in the National registries could be confirmed in hospital records (19), and more confirmed AMI cases were identified in National registries as compared to local registries [22].

#### Experimental procedures

DNA preparation was performed as previously described [23], diluted to 10 ng/ul and arrayed in microtiterplates. PCR, sample preparation and genotyping of the eNOS gene was performed as previously described in detail [24]. Briefly, a fragment overlapping the eNOS polymorphism was amplified by PCR using the primers 5'-CGGCTGGACCCCAGGAAACGG-3' and 5'-TCCAGGGGCACCTCAAGGACC-3'. Template preparation and annealing of sequencing primer (5'-CAGAAGGAAGAGTTCTGGG-3') was performed in a Magnatrix 1200 instrument (Magnetic Biosolution, Stockholm, Sweden) with the standard method and kit provided by the manufacturer, followed by Pyrosequencing analysis on a PSQ™ HS 96 instrument (Pyrosequencing, Uppsala, Sweden).

#### Statistical analysis

To test our primary hypothesis of a synergistic effect between T2DM and endothelial dysfunction due to the eNOS Asp^298 ^allele, we categorised the study population into subjects without diabetes and hypertension; Glu^298^/Glu^298 ^(1), Asp^298^/Glu^298 ^(2), and Asp^298^/Asp^298 ^(3), and into subjects with T2DM; Glu^298^/Glu^298 ^(4), Asp^298^/Glu^298 ^(5), and Asp^298^/Asp^298 ^(6), respectively, thereby generating six categories. The hazard ratios (HR) of AMI morbidity were analysed pair-wise using category 1 as reference.

SPSS Base System for Macintosh 11.0 was used for data analyses and all analyses were sex-specific. Means were adjusted for age and proportions for characteristics of the study population were age-standardised in ten-year intervals using the whole Skara population ≥ 40 years as standard (Table 1). After controlling for that the assumption of proportionality was met, HR were examined by Cox proportional hazard model and expressed with 95 per cent confidence interval. Confounders were accounted for in multiple Cox regression models, by first entering age as covariate, then age, BMI, HDL cholesterol, serum triglycerides, daily smoking (yes/no), and leisure time physical activity. In these models all covariates were entered as continuous variables except for smoking which was dichotomised as described above. Log transformation was used to induce normality in serum triglycerides. Two-way interaction terms were used to explore the interaction between sex the eNOS genotype status (using a recessive and dominant model alternatively) with respect to AMI risk. All tests were two-sided and statistical significance was assumed at p < 0.05.

## Results

Baseline characteristics of the 1202 subjects that were successfully genotyped and included are shown in table 1. The overall genotype distribution was found to be in HW equilibrium (table 1) and in the range of that previously described in a southern Swedish population [24]. T2DM (HR 4.04 [2.61–6.27], p < 0.001) was an independent risk factor for AMI in a multivariate model (adjusted for age and sex), and remained significant when also entering the eNOS Asp^298 ^dichotomized using a recessive model (Asp^298^/Asp^298 ^versus Asp^298^/Glu^298^+ Glu^298^/Glu^298^). Male sex was a statistically significant risk factor for AMI in the population reference group (2.73 [1.21–6.17], p = 0.016), however, not among subjects with T2DM (1.37 [0.89–2.10], p = 0.152).

In order to test the hypothesis that the genotype effect is manifested in the subgroup of patients with T2DM, we analysed the effect of the eNOS Asp^298 ^allele using the subjects with Glu^298^/Glu^298 ^as reference group, stratifying for diabetes, and adjusting for age, BMI, systolic blood pressure, serum triglycerides, HDL -cholesterol, current smoking, and leisure time physical activity. We found a significant risk for AMI associated with the Asp^298^/Asp^298 ^genotype compared to the wild type Glu^298^/Glu^298 ^among patients with T2DM (HR 3.12 CI [1.49–6.56], p = 0.003) but no association between eNOS genotype and AMI in subjects without T2DM (HR 0.45 [0.18–1.11], p = 0.083). When stratifying for sex, we found a significant risk association in women with T2DM (OR 3.56 [1.16–10.90], p = 0.026) (table 2), which could not be fully confirmed in men with T2DM (OR 2.67 [0.96–7.41], p = 0.059). No significant risk associations between AMI and the Asp^298^/Asp^298 ^genotype can be found in subjects without diabetes of either sex. An interaction term between sex and the eNOS genotype dichotomized using the recessive model for the genetic effect was non-significant both in subjects with and without T2DM.

**Table 2:** Hazard ratios of a MI exploring a recessive effect of the Asp 298 polymorphism, with Glu298Glu as reference category, in men and women with T2DM.

	No MI		MI				
**Men**	n = 156	(%)	n = 46	(%)	HR	95%CI	p
*Patients with T2DM*							
*Adjusted for differences in age:*							
Glu298Glu	89	79.5	23	20.5	1.00		
Glu298Asp	57	76.0	18	24.0	1.13	0.61.2.09	0.705
Asp298Asp	10	66.7	5	33.3	2.54	0.95–6.79	0.064
*Adjusted for differences in age, smoking, BMI, systolic blood pressure, HDL-cholesterol, serum triglycerides, and LTPA:*							
Glu298Glu					1.00		
Glu298Asp					1.25	0.65–2.39	0.499
Asp298Asp					2.67	0.96–7.41	0.059

	No MI		MI				
**Women**	n = 162	(%)	n = 39	(%)	HR	95%CI	p

*Patients with T2DM*							
*Adjusted for differences in age:*							
Glu298Glu	90	81.8	20	18.2	1.00		
Glu298Asp	64	82.1	14	17.9	0.92	0.46–1.84	0.808
Asp298Asp	8	61.5	5	38.5	2.97	1.11–7.95	0.030
*Adjusted for differences in age, smoking, BMI, systolic blood pressure, HDL-cholesterol, serum triglycerides, and LTPA:*							
Glu298Glu					1.00		
Glu298Asp					1.11	0.52–2.36	0.790
Asp298Asp					3.56	1.16–10.90	0.026

Hazard ratios (HR) with 95% confidence intervals (955CI) were estimated using multivariate Cox proportional hazard models. BMI; body mass index. LTPA; leisure time physical activity.

**Figure 1:**
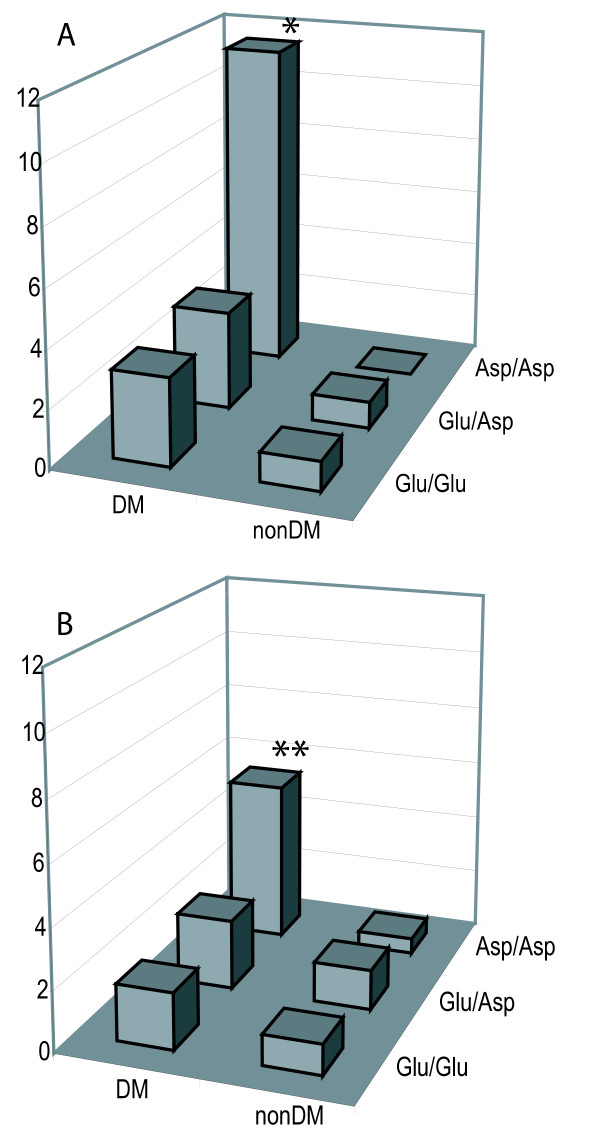
**The risk of myocardial infarction as compared to the reference group of wild type non-diabetics (adjusted for age, sex, smoking, BMI, systolic blood pressure, HDL-cholesterol, serum triglycerides, and LTPA) in women (A) or men (B).** Homozyogosity for the eNOS Asp^298 ^allele is a significant risk factor of greater magnitude in women than in men. * significant (p = 0.001). **significant (p = 0.003)

In order to test the hypothesis of synergism between T2DM and the Asp^298 ^allele in predisposing to AMI, we stratified the study population by genotype (Glu^298^/Glu^298^, Asp^298^/Glu^298^, Asp^298^/Asp^298^) and T2DM (DM +/-), thereby generating six groups. We compared the six groups using as reference group the subjects without diabetes and hypertension who were wild type homozygotes. The synergistic pattern is illustrated in figure 1. Among subjects without diabetes and hypertension, neither heterozygotes nor homozygotes for the Asp^298 ^allele had an increased risk for AMI compared to the reference group. The three groups of patients with T2DM all had a significantly increased risk for AMI compared to the reference group, but in the homozygotes for the Asp^298 ^allele, this risk was markedly higher (OR 7.05 CI [2.96–16.82], p < 0.001). When stratifying for sex, this pattern seemed stronger in women (OR 11.46 [2.63–49.97], p < 0.001) than in men (OR 5.40 [1.77–16.52] p = 0.003) (all values adjusted for differences in age, LTPA, smoking, BMI, systolic blood pressure, HDL-cholesterol, and serum triglycerides).

## Discussion

Our findings show that T2DM is a significant independent risk factor for AMI in both men and women, and that in a subgroup of patients with T2DM, homozygosity for the eNOS Asp^298 ^allele is a significant risk factor for AMI. The eNOS polymorphism has been investigated as a risk factor for cardiovascular disease in numerous studies, and a meta analysis including 23 028 patients showed that homozygosity for the Asp^298 ^allele is associated with a moderately increased risk for IHD [10]. T2DM and insulin resistance are characterized by increased prevalence of cardiovascular disease, and although endothelial dysfunction has been linked to T2DM [25,26] few studies have assessed a possible link between genetic variation in the eNOS gene and the occurrence of T2DM disease. Conflicting results have been reported [12,27-29], however in a large prospective study that included over 24 000 subjects, Conen *et al *[29] found no evidence for an association between the occurrence of T2DM and any single genetic polymorphism or haplotype in the eNOS gene locus. Together with our data, this would suggest that the eNOS pathway is not an important pathological bottleneck in development of T2DM, but has a significant role in processes leading to AMI in patients with already established T2DM.

The comparatively high HR:s we here find restricted to the subgroup of patients with T2DM follow a pattern from previous findings where significant HR:s were detectable in defined subgroups carrying additional atherosclerosis risk factors [30] but not in the overall materials. Together, it supports a hypothesis of that when common factors are combined, each with moderate effect on the NO pathway, synergistic mechanisms could result in a significantly increased risk.

In a previous study, a significant modulation of NO formation in plasma due to genetic variations in eNOS gene could not be found when individual polymorphisms (including the Glu298Asp) were analysed, but was detected when these polymorphisms were combined into haplotypes in the analysis [31]. This suggests that a combined effect of different genetic variants in the eNOS gene also could exist, and it is possible that an extended haplotype analysis covering other polymorphisms in the eNOS gene could have resulted in an even stronger association between AMI and a specific eNOS haplotype in T2DM subject than we show here for the Glu298Asp polymorphism.

It has previously been shown that pre-menopausal women have a lower risk of developing cardiovascular disease than similarly aged men but that this female advantage is reversed in persons with diabetes [17,32]. The effect of T2DM on fatal AMI appears significantly stronger in women than in men independent of other major cardiovascular risk factors [17]. Our results indicate that the eNOS pathway could be more important in women than in men regarding the T2DM-associated MI risk, and may offer part of an explanation for the higher risk increase observed when women get T2DM, compared to men [17,32]. The reason for a sex difference remains unclear but known mechanisms based on hormonal differences make plausible hypotheses.

Two factors that modulate the development of cardiovascular diseases are HDL and estradiol [33,34], both which also can promote the stimulation of eNOS and generation of nitric oxide [35,36]. A large part of the protective effect of HDL in cardiovascular disease is attributed to its role in reverse cholesterol transport [34]. Interestingly, in both mice and humans, female HDL but not male HDL has been shown to stimulate eNOS [37]. This effect was restricted to pre-menopausal HDL and shown to be due to estradiol associated with the lipoprotein [37]. This inability of postmenopausal HDL to stimulate eNOS [37] agrees with a more severe endothelial dysfunction in women after menopause and the increased cardiovascular disease risk [38]. If eNOS stimulation rely on partly different mechanisms in men and women under normal physiological conditions, women after menopause would be more susceptible/vulnerable to any additional factors that impair eNOS function and NO bioavailability than men, and this could offer a putative explanation to why the combined effect of T2DM and the Asp^298 ^allele is stronger in women than in men in our survey. The majority of women in our study were older than 50 years at baseline, and can reasonably be presumed to be past menopause, however definite data on menopause status was not available. HDL from postmenopausal women receiving estrogen replacement therapy has been shown to stimulate eNOS [37] and initial epidemiological studies indicated a protective effect against coronary artery disease [39]. However, two large controlled studies have shown that postmenopausal hormone therapy actually can increase the risk of cardiovascular disease [40,41]. Although exact data is not available in our study regarding the use of hormone replacement therapy (HRT), it was generally low in this population during the time of the study, and HRT use is therefore not likely to influence our results.

The study has some limitations. We have not followed the health status of the control population regarding diabetic status after baseline surveillance/inclusion. However, this misclassification may rather underestimate the risk here observed, since patients with T2DM may exist in the population control material. Stratification into T2DM, genotype and sex results in small numbers within groups. However, we still find significant associations and a consistent pattern when exploring biologically plausible mechanisms supported by previously published *in vitro *and *in vivo *data. A strength of this study is the availability of data that make it possible to adjust for several important confounding risk factors. However, although we adjust for major possible confounders such as age, LTPA, smoking, BMI, systolic blood pressure, HDL-cholesterol, and serum triglycerides, others may still not be accounted for. Previous results have indicated that effects of statins can be modulated by eNOS polymorphism, but unfortunately we have no information on the use of statins at baseline. However, these surveys were conducted 1992–1994, and the use of statins was generally low in Swedish primary care at that time. We choose not to include individuals with hypertension in the reference group to allow for testing specifically the role of the eNOS polymorphism in predisposing to acute myocardial infarction in type 2 diabetics. Moreover, to include subjects with hypertension into the population reference group would have introduced a misclassification, as more than 50% of subjects with hypertension also have diabetes. More strengths of the study are that the study population is of a reasonably large size and with a high participation rate, and furthermore that it is a population-based study where patients are consecutively recruited in primary care and not through specialist clinics, and that the reference subjects come from the population in the same community. Together, this makes it probable that our findings reflect a risk association that is general to patients with T2DM.

## Conclusion

We show a strong independent association of the eNOS genotype with myocardial infarction in patients with T2DM and which is emphasized in females. This supports the role of eNOS as an important pathological bottleneck in cardiovascular disease and a possible target of intervention. Secondary prevention that involves factors which influence eNOS function and NO bioavailability may be of even more benefit among patients with T2DM, especially in women. The observed sex difference also suggest that more aggressive or pre-emptive pharmacological treatment with commonly used drugs that promote eNOS activity or NO bioavailability (statins, angiotensinogen inhibitors, AT-1 blockers, folate, antioxidants) [2,8,42] could be considered in women with T2DM. Whether there is a potential beneficial and sex-dependent effect of treatment with direct or indirect NO donors for preventing cardiovascular disease in patients with T2DM could be of interest to explore further [7,8].

## Abbreviations

AMI: acute myocardial infarction; Asp^298^: Aspartate at position 298; CAD: Coronary artery disesase; Glu^298^: Glutamate at position 298; BMI: body mass index; eNOS: Endothelial nitric oxide synthase; HDL: high density lipoprotein; HRT: hormon replacement therapy; LTPA: Leisure time physical activity; NO: Nitric oxide; HR: hazard ratios.

## Competing interests

The authors declare that they have no competing interests.

## Authors' contributions

JO conceived and designed the current study, was in charge of the molecular genetic analyses, participated in the analyses and the interpretation of the data, and wrote the paper. CL participated in preparing and interpreting the data, and in writing the paper. LR initated the Skaraborg project, participated in interpreting the data, and in writing the paper. UL initiated the Skaraborg project, conceived and designed the current study, performed the statistical analyses, participated in interpreting the data, and participated in writing the paper. All authors read and approved the final version.

## Pre-publication history

The pre-publication history for this paper can be accessed here:


